# Diversity in Glycosaminoglycan Binding Amongst hMPV G Protein Lineages

**DOI:** 10.3390/v4123785

**Published:** 2012-12-14

**Authors:** Penelope Adamson, Sutthiwan Thammawat, Gamaliel Muchondo, Tania Sadlon, David Gordon

**Affiliations:** 1 Department of Microbiology and Infectious Diseases, Flinders University, Flinders Medical Centre, Bedford Park, SA 5042, Australia; E-Mails: ant3714@hotmail.com (S.T.); gamaliel.muchondo@health.sa.gov.au (G.M.); tania.sadlon@health.sa.gov.au (T.S.); d.gordon@flinders.edu.au (D.G.); 2 Department of Microbiology and Infectious Diseases, SA Pathology, Flinders Medical Centre, Bedford Park, SA 5042, Australia

**Keywords:** human metapneumovirus (hMPV), Glycosaminoglycan (GAG), G protein, F protein, infectivity

## Abstract

We have previously shown that hMPV G protein (B2 lineage) interacts with cellular glycosaminoglycans (GAGs). In this study we examined subtypes A1, A2 and B1 for this interaction. GAG-dependent infectivity of available hMPV strains was demonstrated using GAG-deficient cells and heparin competition. We expressed the G protein ectodomains from all strains and analysed these by heparin affinity chromatography. In contrast to the B2 lineage, neither the A2 or B1 G proteins bound to heparin. Sequence analysis of these strains indicated that although there was some homology with the B2 heparin-binding domains, there were less positively charged residues, providing a likely explanation for the lack of binding. Although sequence analysis did not demonstrate well defined positively charged domains in G protein of the A1 strain, this protein was able to bind heparin, albeit with a lower affinity than G protein of the B2 strain. These results indicate diversity in GAG interactions between G proteins of different lineages and suggest that the GAG-dependency of all strains may be mediated by interaction with an alternative surface protein, most probably the conserved fusion (F) protein. Analysis of both native and recombinant F protein confirmed that F protein binds heparin, supporting this conclusion.

## 1. Introduction

Human metapneumovirus (hMPV) is responsible for causing serious respiratory illness, most commonly in infants and young children, but also in the elderly and immunocompromised patients [1,2,3,4,5]. hMPV is a member of the genus *Metapneumovirus* within the family *Paramyxoviridae*. Avian pnuemovirus is the only other member of this genus [5]. Respiratory syncytial virus (RSV), a member of the genus *Pneumovirus*, also within the family *Paramyxoviridae*, is the most closely related human pathogen to hMPV and as such they share many of the same symptoms [6,7,8,9]. These range from mild upper respiratory tract disease to severe lower tract diseases such as bronchiolitis and pneumonia [5] and together, hMPV and RSV, are responsible for at least 50% of all respiratory viral infections requiring hospitalisation in children [10,11].

hMPV is an enveloped, single stranded, negative-sense RNA virus which contains 3 envelope glycoproteins, the fusion (F), attachment (G) and the small hydrophobic (SH) protein. It is the organisation of the genome and the lack of non structural genes which separates hMPV from other paramyxoviruses, such as RSV [5,8]. Phylogenetic analysis of the nucleotide sequence of several hMPV genes has shown that hMPV is divided into two major groups, A and B, both of which can be further divided into two minor subgroups, 1 and 2 [12,13,14,15,16]. hMPV resembles other members of the *Paramyxoviridae* family genetically and morphologically, as determined by electron microscopy [17].

Whilst the fusion protein of hMPV is highly conserved, immunogenic and induces protective antibodies, the other surface glycoproteins, G and SH, unlike most other *Paramyxoviridae*, have been shown to be only weakly or negligibly immunogenic [18,19,20,21,22]. The G protein of hMPV is a type II membrane protein consisting of extracellular, transmembrane and intracellular domains [8] and is highly glycosylated [12,23]. It is highly variable, particularly the extracellular domain [13]. This diversity along with its extensive glycosylation probably aids in its evasion of the immune system [12]. The role of the G protein has not been fully elucidated, however previous studies indicate that it likely plays a role in viral attachment [24] and replication [25].

Glycosaminoglycans (GAGs) are linear polysaccharides which are comprised of repeating disaccharide units. The repeat units of GAGs consist of an amino sugar (N-acetylglucosamine or N‑acetylgalactosamine) and uronic acid (glucuronic or iduronic acid) or galactose which are variably sulphated, except hyaluronic acid which lacks sulphate groups [26]. They are covalently attached to core proteins to form proteoglycans which are found in tissue, the extracellular matrix (ECM) and on the inside and the surface of most cell types [26].

A number of viruses, including other paramyxoviruses, have been shown to interact with cell surface GAGs to facilitate cellular attachment and subsequent entry into cells [27,28,29,30]. Past studies of RSV and our previous study of hMPV have shown that infection is markedly or completely inhibited by the presence of soluble GAGs such as heparin, by removing GAGs from the surface of cells enzymatically or with the use of GAG deficient cells [24,31,32].

We have shown previously that attachment of hMPV to cells may be mediated by a G protein-GAG interaction. This initial work was carried out using G protein of the B2 lineage. Due to the high variability of G protein between strains we investigated if this is consistent for all hMPV strains. Using recombinantly expressed G protein we demonstrated that, other that the B2 strain, only the A1 strain binds to GAGs. By truncating the G proteins of the A1 and B2 subtypes, we have further characterised the functional domains within each protein involved in these interactions. Furthermore, using hMPV infected cell lysates and recombinant F protein, we show binding of the F protein to GAGs suggesting that this protein is also involved in virus attachment to the cell surface, and providing an explanation for the GAG-dependency of all hMPV strains.

## 2. Results and Discussion

### 2.1. Susceptibility of hMPV Primary Isolates to Inhibition of Infection by Heparin

To date our investigations have focused on G protein from the hMPV B2 strain. To determine if the infectivity of other strains is also GAG-dependent, we incubated primary isolates of hMPV A2, B1 and B2 strains with and without heparin before inoculation of LLC-MK2 cells. Infectivity was determined using quantitative real time PCR. Primary isolates were used to preclude the possibility that heparin binding was an adaptation of the virus during cell culture. hMPV A1 strain is not represented in these experiments as a primary isolate could not be obtained during the specimen collection period. Infection with all available hMPV strains was markedly or completely inhibited by heparin pre-incubation (Table 1).

**Table 1:** Susceptibility of hMPV primary isolates to inhibition of infection by heparin ¹.

Isolate	Subtype	Ct value ^2^	
		Heparin Pre-treated	Untreated virus
V47041	B2	Negative	24.1
V52283	A2	30.2	16.6
V50569	B1	Negative	13.1

¹ LLC-MK2 cells were inoculated with primary isolates of hMPV, ±50 IU heparin pre-treatment. Infectivity was determined by a quantitative real-time RT-PCR for nucleoprotein gene. ^2^ Results are represented as a cycle threshold (Ct) value. Higher or negative Ct values after heparin pretreatment of the virus indicate reduced viral infectivity.

### 2.2. GAG-Deficient CHO Cells Are Resistant to hMPV Infection

**Figure 1:**
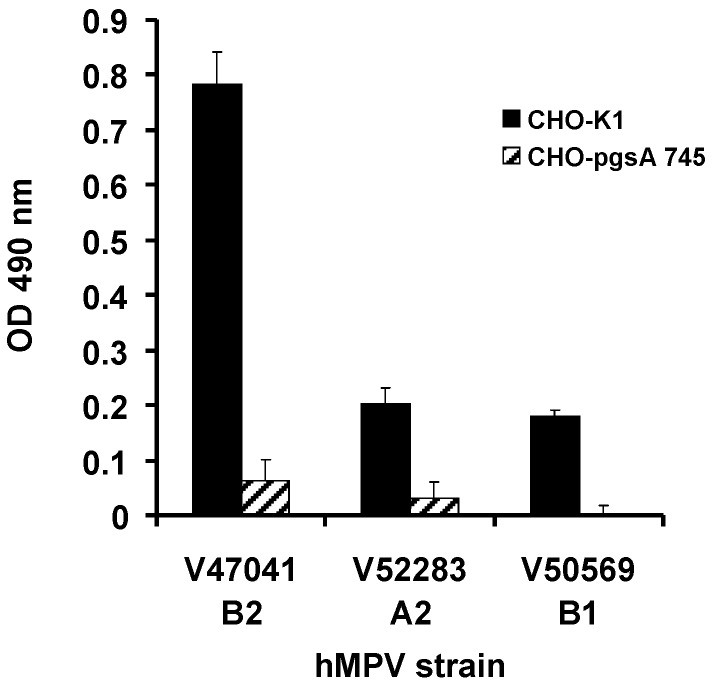
Primary isolates of hMPV utilise GAGs to mediate infection. CHO-K1 and CHO-pgsA 745 cells were inoculated with hMPV PCR positive nasopharyngeal aspirates. LLC-MK2 cells were inoculated with the CHO cell supernatants and infectivity determined by cell ELISA using a hMPV matrix protein mAb. Data represent mean values ± SD of triplicate wells.

As soluble heparin was able to reduce or completely inhibit infection of LLC-MK2 cells, we examined the ability of the 3 primary isolates to infect CHO-K1 and CHO-pgsA 745 cells. CHO-pgsA 745 cells lack xylosyltransferase activity and therefore are deficient in cellular GAGs [33]. The CHO‑K1 and CHO-pgsA cells were incubated for 2 weeks with nasopharyngeal aspirates positive for hMPV by PCR. As the viral titres from CHO cultures were low, the wild type and mutant CHO-K1 cell supernatants were tested for infectious hMPV by subsequent incubation with LLC-MK2 cells followed by cell ELISA. Infectivity of all three strains, hMPV A2, B1 and B2, was dependent on GAGs as GAG deficient CHO-K1 cells had negligible evidence of infection (Figure 1).

### 2.3. Binding of hMPV G Glycoprotein to Immobilised Heparin

**Figure 2:**
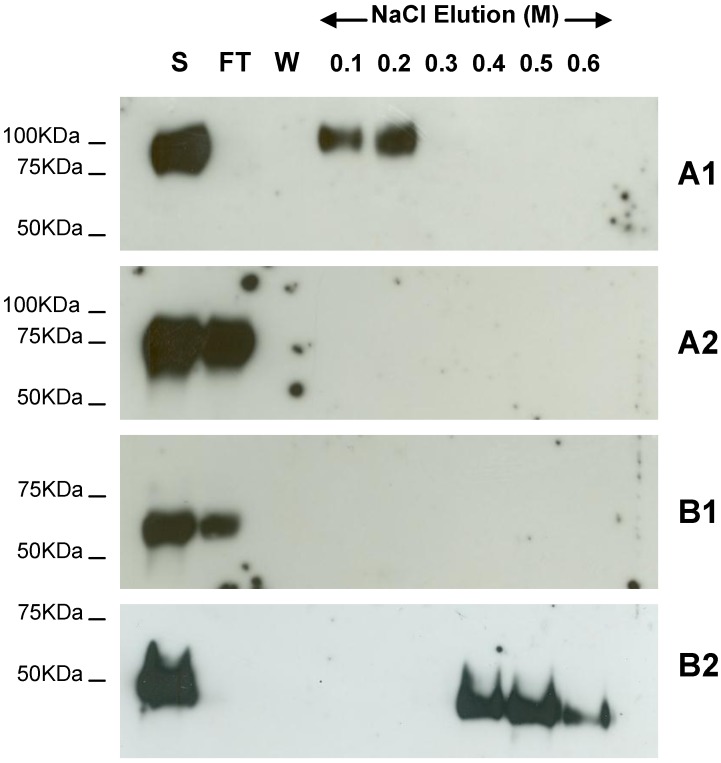
Heparin agarose affinity chromatography of recombinant G ectodomain for A1, A2, B1 and B2 hMPV strains. The start (S), flow through (FT), final wash (W) and salt elution fractions were analyzed by 10% SDS-PAGE under reducing conditions and western blot analysis using anti-c-Myc monoclonal antibody. A1, A2, B1 and B2 indicate the strain type.

Previous studies carried out in our laboratory demonstrated the binding of G protein of hMPV B2 strain to cellular GAGs [24]. To determine if this is consistent for all strains of hMPV, we expressed the extracellular domain of the G protein for the A1, A2 and B1 strains in the yeast *Pichia pastoris* X33. The recombinant B1 G protein migrated at an apparent molecular weight of 60 kDa which is comparable to the recombinant B2 ectodomain, however the apparent molecular weights for the A1 and A2 protein migration were 100 kDa and 75 kDa, respectively (Figure 2). The purified recombinant proteins were applied to heparin agarose columns and after extensive washing were eluted with a stepwise salt gradient. The recombinant G ectodomains of hMPV strains B2 and A1 bound to the heparin column while strains A2 and B1 did not (Figure 2). The recombinant hMPV B2 G protein appears to have a higher affinity for heparin than the recombinant hMPV A1 G protein as it requires a higher salt concentration for elution.

### 2.4. Functional Domains in hMPV G A1 Protein Involved in GAG Interactions

**Figure 3:**
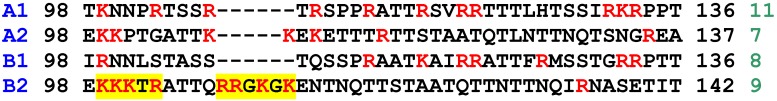
Comparison of the predicted amino acid sequence for representatives of each strain of hMPV G protein (residues 98–136/137/142 of the extracellular domain). Strains are shown in blue and number of positively charged residues (shown in red in the sequence) is indicated in green at the end of each sequence. The yellow highlights in the B2 sequence indicate the previously identified heparin binding domains [24].

We have previously identified 2 adjacent regions of highly charged amino acids within the extracellular region of the B2 G protein which are important for heparin binding [24]. These domains are less well defined in other hMPV strains (Figure 3) and certainly appear to be lacking in the A1 and B1 strains. Despite this, the A1 strain G protein still interacts with heparin, perhaps due to the high number of positively charged residues adjacent to this region.

**Figure 4:**
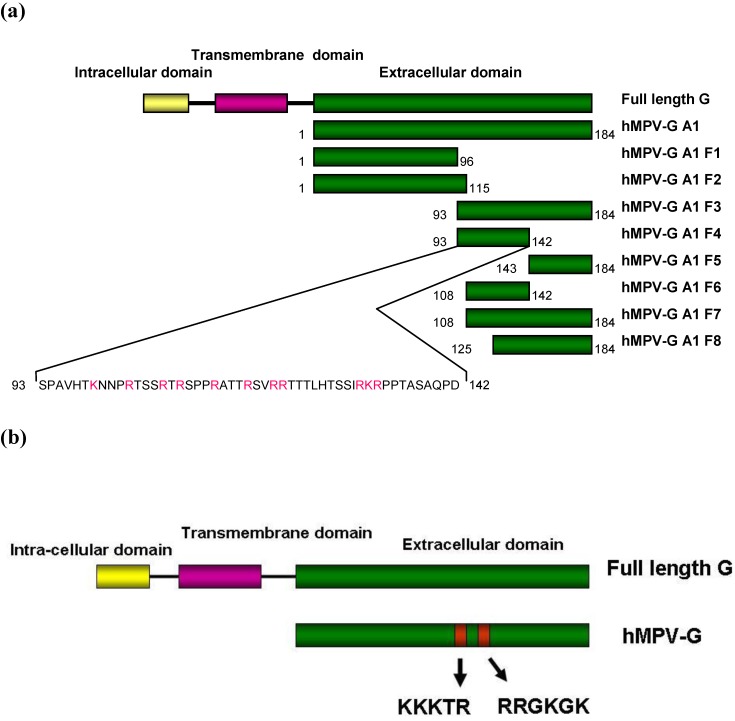
A schematic diagram of recombinant hMPV G protein from (**a**) the A1 strain and the fragments produced and (**b**) the B2 strain. hMPV‑G A1 F1, F2, F3, F4, F5, F6, F7 and F8 indicate the 8 fragments of hMPV G A1 strain that were engineered. The sequence of the smallest fragment that binds to heparin (hMPV-G A1 F4) is shown with the positively charged residues in red. The red boxes represent the clusters of positively charged amino acids that are considered potential heparin binding sites.

**Figure 5:**
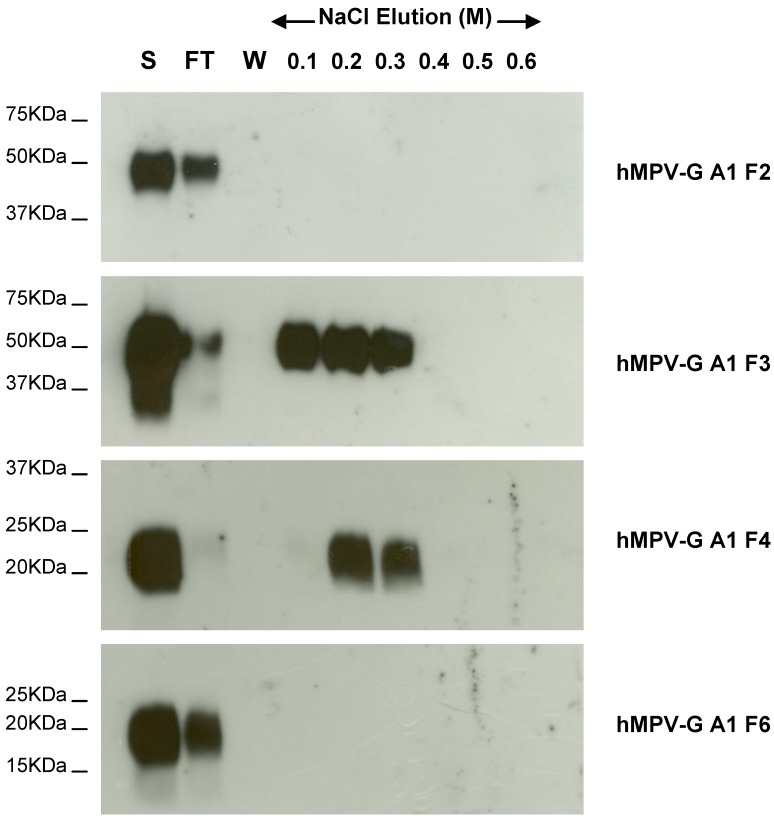
Heparin agarose affinity chromatography of recombinant F2, F3, F4, and F6 fragments of the hMPV G A1 strain ectodomain. The start (S), flow through (FT), final wash (W) and salt elution fractions were analysed by 10%–14% SDS-PAGE under reducing conditions and western blot analysis using anti-c-Myc MAb.

To identify the regions of A1 strain G protein involved in binding to heparin, we constructed 8 fragments of the hMPV G A1 ectodomain (Figure 4a), particularly targeting the region previously shown to be important for binding in hMPV G B2 strain (Figure 4b). These recombinant truncations migrated at sizes on SDS PAGE which varied depending on the length of the hMPV G (A1) fragment expressed. The purified hMPV G A1 fragments were applied to a heparin agarose column and following extensive washing were eluted with a stepwise salt gradient. hMPV-G A1 F3 and F4, but none of the other fragments, bound to heparin agarose (Figure 5, some data not shown). hMPV-G A1 F4 (residues 93–142 of the extracellular domain) is the smallest fragment which binds heparin. A smaller fragment, hMPV-G A1 F6 (residues 108–142), was unable to bind heparin, which indicates that residues 93–108 are crucial for the interaction of hMPV-G A1 F4 with heparin. However, since the non-binding hMPV-G A1 F2 (residues 1–115) also incorporates residues 93–108, there must be additional residues required for the heparin-G protein interaction between residues 115 and 142. These results indicate that the region of GAG binding in hMPV G A1 is similar to that identified in hMPV G B2 despite the fact that there are no well defined positively charged clusters in this protein.

### 2.5. Binding of hMPV F Protein to Immobilised Heparin

**Figure 6:**
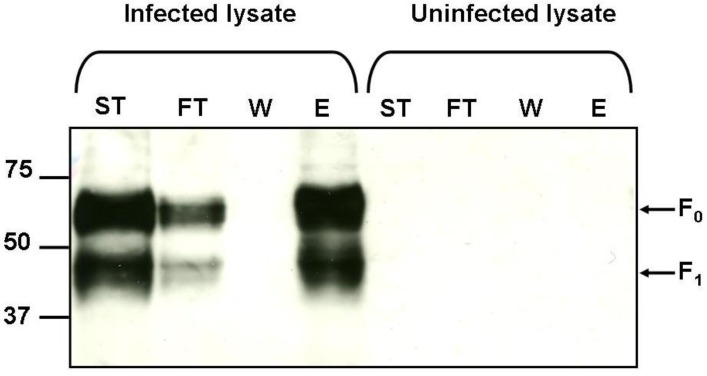
Binding of native hMPV F protein to heparin. Start material (ST), flow through (FT), wash (W) and 2M NaCl elution (E) fractions were analysed by 10% SDS-PAGE under reducing conditions and western blot analysis using anti-hMPV F antibody. Arrows indicate bands corresponding to the predicted sizes of full length precursor hMPV-F (F_0_) and the cleavage fragment hMPV F_1_. Molecular weight markers are shown in kDa.

The results of the infection experiments indicate that infection with the available strains of hMPV is dependent on the presence of GAGs; however not all of the hMPV G recombinant proteins bind to immobilised heparin (Figure 2). This implies that there are interactions occurring between GAGs and other surface exposed proteins on hMPV. An alternative to the attachment (G) protein is the fusion (F) protein which is highly conserved across all strains of hMPV. Fusion protein binding to GAGs was demonstrated by incubating hMPV infected cell lysates with heparin agarose and elution with salt (Figure 6). Both the precursor F protein, F_0_, and the biologically active cleaved F_1_ protein bound to heparin. No proteins were detected when uninfected cell lysate was subjected to heparin affinity precipitation (Figure 6).

**Figure 7:**
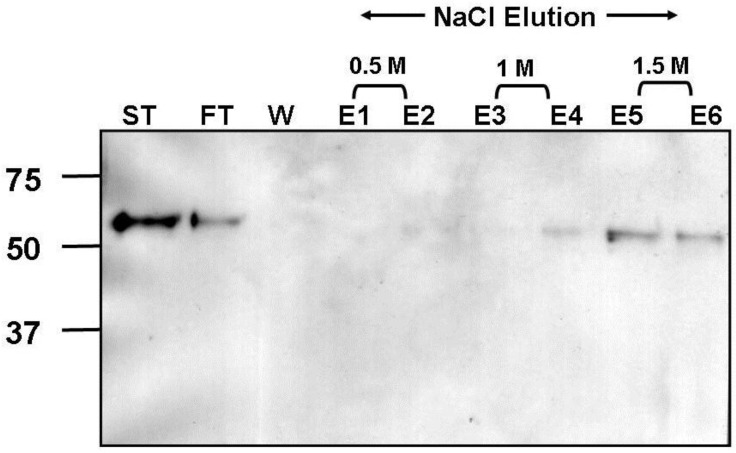
Heparin agarose affinity chromotography of recombinant soluble hMPV-F protein. Start material (ST), flow through (FT), wash (W) and elution (E) fractions, with 0.5M NaCl (E1 & E2), 1M NaCl (E3 & E4) and 1.5M NaCl (E5 & E6), were analysed by 10% SDS-PAGE under reducing conditions and western blot analysis using anti-hMPV F antibody. Molecular weight markers are shown in kDa.

Additionally, the extracellular domain of F protein was cloned and expressed in a mammalian expression system. This resulted in a recombinant protein which migrated on SDS PAGE according to the predicted molecular weight of the uncleaved precursor form (F_0_). Binding of soluble recombinant F protein to GAGs was investigated by heparin affinity chromatography. Recombinant hMPV-F protein bound to heparin with high affinity as demonstrated by protein elution with a high salt concentration (Figure 7).

## 3. Experimental Section

### 3.1. Cells and Viruses

The rhesus monkey kidney cells (LLC-MK2) and the human epithelial tumour cell line (HEp-2) were grown in medium 199 (Invitrogen, Carlsbad, CA) supplemented with 10% foetal bovine serum (FBS). Wild type Chinese hamster ovary cells (CHO-K1) and GAG-deficient CHO-pgsA745 cells [33] were grown in Hams F12 medium (Invitrogen) supplemented with 10% FBS.

The A2 (V52283), B1 (V50569) and B2 (V47041) strains of hMPV were isolated from clinical samples by the Virology Laboratory, Flinders Medical Centre (FMC). These samples were positive only for hMPV and were not coinfected with influenza A, influenza B, RSV, adenovirus or parainfluenza 1, 2 or 3. Stocks of hMPV were prepared by inoculating LLC-MK2 cells with the hMPV strains and incubating for 14 to 21 days at 37 °C in 5% CO_2_. Stocks were stored at −70 °C until use. RNA extracted from the hMPV A1 strain was kindly provided to us by Dr. I. Mackay (Queensland Paediatric Infectious Diseases Laboratory, Sir Albert Sakzewski Virus Research Centre, Royal Children’s Hospital, Queensland, Australia). hMPV strain subtyping was based on the Ishiguro classification [14].

### 3.2. Heparin Inhibition of hMPV Infection

Primary isolates of hMPV, A2, B1 and B2 strains (A1 was not available as it was not detected in South Australia during the collection period for these isolates), were pre-treated with or without heparin (50 IU/mL heparin) at 37 °C for 30 min before inoculation of LLC-MK2 cells. The cultures were incubated for 14 days with the maintenance medium changed every 3 days. Infectivity was determined after 14 days by a quantitative real-time RT-PCR for a 163 bp region of the nucleoprotein gene of hMPV [34]. Viral RNA extracted from the cultures was reverse transcribed using 50 U MMLV reverse transcriptase and 1 μM NL-N-reverse following the manufacturer’s protocol. Five μL cDNA was amplified using a Rotor Gene RG-3000 (Corbett Research, Sydney, Australia) in a 25 μL reaction containing 1 × PCR buffer, 5 mM MgCl_2_, 200 μM dNTPs, 0.4 μM NL-N-forward, 0.2 μM NL‑N‑reverse, 0.1 μM NL-N-probe and 0.75 U HotStar Taq polymerase (QIAGEN) with a 15 min Taq activation at 95 °C followed by 55 cycles of 10 s at 95 °C, 15 s at 55 °C and 15 s at 72 °C.

### 3.3. GAG Dependence of hMPV Primary Isolates

CHO-K1 and CHO-pgsA cells were incubated with 200 μL of the primary hMPV isolates for strains A2, B1 and B2 in 1 mL CHO maintenance medium in 14 mL conical tubes (Cellstar, Greiner Bio‑one, Frickenhausen, Germany) at 37 °C, 5% CO_2_ for 14 days with the medium changed every 3 days. Two hundred microlitres of the culture supernatants, collected on day 14, were used to inoculate LLC-MK2 cells in 96 well tissue culture plates (Linbro, ICN Biomedicals, Aurora, OH, USA). After incubation for 2 h in 37 °C, 5% CO_2_ the inoculum was replaced with 200 μL maintenance medium and incubated for 7 days at 37 °C, 5% CO_2_. 

Infectivity was assessed using a cell based enzyme linked immunosorbent assay (ELISA) [24]. Briefly, confluent monolayers of Hep-2 cells in 96 well plates were inoculated with day 7 LLC-MK2 cell supernatants and incubated for 2 h at 37 °C, 5% CO_2_. Unbound virus was removed by washing with medium 199 and the cells were cultured in medium 199 containing 1 μg/mL trypsin at 37 °C, 5% CO_2_. After 48 h the cells were fixed with 1% paraformaldehyde in phosphate buffered saline (PBS) for 30 min at room temperature. Cells were washed twice with PBS, permeabilised with 0.02% Triton X-100/PBS for 30 min at 4 °C and then washed twice with PBS. The cells were blocked with 5% skim milk/PBS for 1 h before incubation with 1/320 (v/v) hMPV matrix protein monoclonal antibody (Chemicon, Temecula, CA, USA) in 0.5% Tween 20/PBS followed by 1/10,000 (v/v) horseradish peroxidase (HRP) conjugated sheep anti-mouse immunoglobulin G (Chemicon) in 0.5% Tween 20/PBS. All incubations were 1 h at 37 °C and the wells were washed four times with PBS after each step. The wells were developed by incubating with *O*-phenylenediamine substrate (Sigma, St. Louis, MO, USA) for 30 min. Reactions were stopped with 1N H_2_SO_4_ and the absorbance at 490 nm was determined. Wells were inoculated in triplicate and each experiment carried out at least twice.

### 3.4. Construction and Expression of Recombinant hMPV G Proteins

The ectodomains of A1, A2 and B1 were cloned and expressed for this study using similar methods as that described for the construction and expression of the extracellular domain of the G protein of the B2 strain of hMPV [24]. Briefly, RNA was extracted from hMPV (subtype A2 and B1) infected LLC‑MK2 cells using the QIAamp Viral RNA purification kit (QIAGEN, Hilden, Germany) following the manufacturer’s protocol. cDNA was synthesised using 500 μM GB1R or GA2R, as appropriate, or 50 ng random primer (Promega, Madison, WI, USA) for the A1 strain, using 200 U Moloney murine leukaemia virus (MMLV) reverse transcriptase (Invitrogen) according to the manufacturer’s conditions. The ectodomain of hMPV G protein was amplified using 0.8 μM forward and reverse primers (Table 2) for individual strains of hMPV or the region of the G protein to be expressed. PCR was carried out in a reaction containing 1 × PCR buffer, 1 × Q-Solution, 1.5 mM MgCl_2_, 400 μM dNTPs and 0.75 U HotStar Taq Polymerase (QIAGEN) and 5 μL cDNA. Forward and reverse primers incorporated restriction sites compatible with those in the multiple cloning site of the pPICZαA plasmid (Invitrogen) as underlined in Table 2. This allowed cloning of the PCR products downstream of the yeast α factor signal sequence, resulting in the secretion of the expressed protein into the growth medium. The PCR products were cloned in frame with the C-terminal tags, c-Myc and 6 × HIS, for ease of detection and purification. Following transformation into *Pichia pastoris* X33 yeast, expression of the soluble recombinant protein was induced with methanol over 3–4 days. The hMPV G proteins were purified using immobilised metal affinity chromatography (IMAC) over HP fast flow chelating columns (GE Life Sciences, Uppsala, Sweden) loaded with nickel chloride. The sequence of each construct was verified prior to transformation into *P. pastoris* X33.

### 3.5. Heparin Agarose Affinity Chromatography

Heparin agarose chromatography was carried out in 50 mM sodium phosphate buffer pH 7.4 (PB) using 1 mL heparin agarose columns (Pierce Chemical Corporation, Rockford, IL, USA and Sigma). Recombinant proteins were dialysed into phosphate buffer overnight and then passed over the column 4–6 times. The columns were extensively washed with phosphate buffer and then eluted in a step-wise NaCl gradient. Start, flow-through, wash and elutions were all analysed using SDS-PAGE and western blotting.

**Table 2:** Primers used to amplify the extracellular region of the hMPV strains A1, A2, B1 and B2, hMPV-G A1 strain fragments and the hMPV F extracellular region. Restriction sites incorporated for ease of cloning are underlined; EcoRI (GAATTC), MluI (ACGCGT) and XbaI (TCTAGA). Bases incorporated to remain in frame with the C-terminal tags are shown in italics.

Primer Name	hMPV Strains	Primer Sequence
GB2F	B2	5'-GGGGAATTCGATCATGCAACATTAAGAAACATG-3'
GB2R	B2	5'-GGGTCTAGA*GC*TCCTGCACCTCYCCGTGCAT-3'
GB1F	B1	5'-AGAATTCGAATCAGAACATCACACCAG-3'
GB1R	B1	5'-ATCTAGA*GC*TGTGCTTGCAGATGCCTG-3'
GA2F	A2	5'-AGAATTCGATTATGCAACATTAAAAAACATG-3'
GA2R	A2	5'-ATCTAGA*GC*ACTACTTAGAGAAGATGTGTC-3'
GA1F	A1	5'-AGAATTCAACTATAMAATGCAARAAAACTCCGA-3'
GA1R	A1	5'-TTCTAGA*GC*ACTAGTTTGGTTGTATGTTGTTGA-3'
GA1F2	A1	5'-AGAATTCAGCCCAGCAGTCCACACAAAAAAC-3'
GA1R1	A1	5'-TTCTAGA*TC*GACTGCTGGGCTTGTCTTTGTTC-3'
GA1R2	A1	5'-TTCTAGA*TC*TGTTGTTGCCCGTGGTGGGGAAC-3'
GA1F3	A1	5'-AGAATTCGACAGCAGCGCAACAATCC-3'
GA1F4	A1	5'-AGAATTCACACGTTCCCCACCACG-3'
GA1F5	A1	5'-AGAATTCCTCCACACAAGCAGCATAAG-3'
GA1R4	A1	5'-TTCTAGA*GC*GTCTGGTTGGGCTGATGC-3'
FMPV-for	B2	5'-GGGACGCGTCTTAAAGAGAGCTAYYTAGAAG-3'
FMPV-r2B	B2	5'-GGGTCTAGAGCRCCAGTGTTTCCTTTTTCTGC-3'

### 3.6. Detection of hMPV G Protein by Western Blot

Proteins were separated by 10%–14% SDS PAGE (depending on the expected size of the protein) under reducing conditions and transferred onto Amersham^TM^ Hybond^TM^-ECL membrane (GE Healthcare). Membranes were blocked with 5% skim milk/PBS and incubated with 1/5 (v/v) anti‑c-Myc monoclonal antibody (from in-house hybridoma cell line 9E10) followed by 1/2,000 (v/v) HRP conjugated sheep anti-mouse immunoglobulin G. The proteins were visualised using enhanced chemiluminescence (ECL).

### 3.7. Heparin Binding Studies Using hMPV Infected Cell Lysates

Monolayers of LLC-MK2 cells were infected with hMPV (over 10 days) or virus/Chlamydia transport medium (VCTM), as a “mock” inoculum control. Cells were washed twice with PBS and lysed with 250 μL 60 mM n-octyl β-B-glucopyranoside (Sigma), 1 × protease inhibitor cocktail tablet (Roche, Mannheim, Germany) in PBS. After 20 min on ice, cell debris was removed by centrifugation at 13,000 × *g* for 15 min at 4 °C and the supernatant collected. Infected and uninfected cell lysates were diluted 1:2 (v/v) in PB and incubated with heparin agarose, washed and eluted with salt. Fractions were analysed by SDS-PAGE and western blotting.

### 3.8. Detection of hMPV F Protein by Western Blot

Proteins were separated by 10% SDS PAGE under reducing conditions and transferred onto Amersham^TM^ Hybond^TM^-ECL membrane. Membranes were blocked with 5% skim milk/PBS and incubated with 1/2,000 (v/v) hamster polyclonal anti-hMPV F antibody (MedImmune, Gaithersburg, MD, USA) followed by 1/2,000 (v/v) HRP conjugated goat anti-Armenian hamster immunoglobulin (Rockland Immunochemicals, Gilbertsville, PA, USA). The proteins were visualised using ECL.

### 3.9. Construction and Expression of hMPV F Protein

The extracellular domain of the F gene of hMPV (B2 strain) was amplified as described previously using the primers shown in Table 2 and amplification conditions as described for amplification of hMPV G protein. The PCR product was cloned into the mammalian expression vector, BSRαEN [35] downstream of the factor H (fH) signal sequence. The hMPV F/BSRαEN construct was verified by sequencing. CHO-K1 cells were transiently transfected with hMPV F/BSRαEN using Lipofectamine 2000 (Invitrogen) then grown in OPTI-MEM (Invitrogen) for 72 h. Supernatant was harvested and analysed for protein expression using SDS PAGE and western blotting with anti-hMPV F antibody.

## 4. Conclusions

In this study we investigated whether sequence variability between different strains of hMPV G protein would affect the ability of their attachment proteins to interact with GAGs. Although infection with all strains tested (A2, B1 and B2) was inhibited by soluble heparin and required cell surface GAGs for infection, only recombinant G proteins from the A1 and B2 strains bound heparin agarose. Some viruses, including Sindbis and Ross River virus have been shown to adapt during cell culture to bind to heparin [36,37,38]. Sequence analysis of these serially cultured viruses, show the introduction of amino acid substitutions which are likely to create heparin binding domains to better adapt to cell culture and to expand the host range of the viruses. However, this may abrogate the ability of these viruses to retain their pathogenicity as has been shown in culture-adapted Sindbis virus [38]. We confirmed that the GAG dependency of each hMPV strain was not an artifact of serial cell culture passage by using non-passaged primary isolates for these experiments and also by sequencing the G gene of a series of primary isolates (data not shown).

We observed differences in the apparent molecular weight of the G proteins expressed for each strain. The proteins expressed for the B1 and B2 strains migrated on SDS PAGE at apparent molecular weights of approximately 60 kDa and 50 kDa, respectively, however A1 and A2 recombinant G protein migrated at approximately 100 kDa and 75 kDa, respectively. This could be due to varying lengths of the hMPV G ectodomains expressed and/or differences in glycosylation between strains. It has been demonstrated that differential glycosylation of RSV G protein, dependent on the cell line of origin, results in changes in electrophoretic mobility on SDS PAGE [39]. Yeast expression systems, such as *Saccharomyces cerevisiae*, are known to hyperglycosylate secreted proteins, however this is less likely in glycoproteins expressed by *Pichia pastoris* which are thought to resemble those of higher eukaryotes [40].

The interactions between hMPV G protein and GAGs are most likely mediated by electrostatic interactions between positively charged residues in the G ectodomain and negatively charged sulphates on the GAGs rather than at specific recognition sites. It appears from our results that hMPV B2 G protein has a higher affinity for heparin than hMPV A1 G protein. This could be due to the availability of positively charged residues on the protein surface after folding, the number of positively charged residues or the spacing of the residues. The conformation of Kaposi’s sarcoma-associated herpesvirus complement control protein (KCP) is important in the formation of a strong positive patch on the KCP which is then available to bind to heparin. Using docking simulations, Mark *et al.* [41] showed that the extended conformation of this protein presented a more favourable docking site than could be found in the bent conformation. Differences in conformation of the G protein in each strain, due to high sequence variability, may explain the reduced affinity for heparin of hMPV A1 G protein compared to G protein of the B2 strain. To investigate regions of positively charged residues we produced a series of truncations of hMPV G (A1) strain which were tested for binding to heparin agarose. The results indicated a region between residues 93 and 142 may be responsible for G protein-GAG interactions. This domain contained a greater concentration of positively charged residues and is not dissimilar to the region identified for the B2 strain [24]. When the amino acid sequence of G protein from each hMPV strain is compared (Figure 3), GAG-binding strains had a higher number of positively charged residues in the tentative binding region (residues 98–136/137/142). The increased affinity of the G protein of the B2 strain is probably not due to the total number of positively charged residues, as the A1 strain has more in this region, but more likely the clustering of the residues seen in the B2 strain. Consensus motifs, XBBXBX, XBBBXXBX and TXXBXXTBXXXTBB (where B is the probability of basic residues, X is an uncharged hydrophobic residue and T is an amino acid in a turn) have been proposed [42,43] to mediate GAG recognition however these do not hold true for every heparin binding protein, including factor H. This suggests that the orientation and spacing of amino acids, as described by Margalit *et al.* [44], and the local absence of a negative charge are important. Although it is apparent from our previous study [24] that hMPV infection is inhibited by a wide range of GAGs, other viruses, such as RSV, Dengue virus and yellow fever virus, have a far more limited repertoire of cell surface GAGs [45,46]. It may be possible that different strains of hMPV are also this discriminatory and hence do not bind heparin, although our infection experiments suggest that this is unlikely. A complex interaction between the number and clustering of charged residues is ultimately likely to determine GAG-binding characteristics. Further mutagenesis of the A1 strain or engineering additional positively charged residues into this region within the non-binding strains may provide a greater insight into the residues involved.

We have shown that the dependency of hMPV infection on GAGs is not explained by a common interaction of G protein with GAGs. There is debate that the G protein of hMPV is not essential for virus infectivity. Replication of a mutant hMPV virus which was lacking the G protein was shown to be as efficient in cell culture as wild type virus, however replication *in vivo* was markedly reduced [25,47]. Similarly this has been investigated for RSV [48]; however the effect of deleting RSV G protein appears to be cell specific [49] and replication of mutant RSV in the respiratory tract of mice is highly restricted. We have shown in this study, and previously, that both hMPV infectivity and G protein binding can be mediated by GAGs, however it is unlikely that the mechanism of infection is solely reliant on the ability of G protein to bind GAGs. There is evidence that some viruses recognise more than one receptor to gain entry to target host cells [50,51]. Wickham *et al.* [51] described the use of multiple receptors in adenovirus which firstly binds to the cell with a high affinity interaction through the fibre protein and then to integrins via RGD sequences present in the penton base. Another example is the cascade of interactions involved in the entry of HSV into cells which involves several virion glycoproteins, some of which interact with heparin sulphate [52,53,54,55,56,57]. Our results indicate a mechanism similar to that described for adenovirus. The inhibition of infection of the two strains not shown to bind heparin agarose via their attachment proteins, can be explained by the ability of the highly conserved F protein to bind to heparin. We showed that F protein, either in its native form, as part of a hMPV infected cell lysate, or expressed recombinantly, was able to bind to heparin with high affinity. This indicates that the GAG dependency of all hMPV strains for infectivity may be mediated by F protein-GAG interactions. These results have recently been confirmed by Chang *et al.* [58] who demonstrated that interactions between the hMPV F protein (of the A2 subtype) and the glycosaminoglycan heparan sulphate was essential for efficient binding of virus to cell surfaces and is most likely the initial step in virus binding and infection. Virus attachment and entry appears to require a combination of GAG binding with, or as a precursor to, other interactions, including integrin binding. Recently an integrin binding recognition Arg-Gly-Asp (RGD) sequence was identified in the F protein of hMPV which is conserved across all strains [19]. The F protein was shown to interact with the integrin αvβ1 and this was suggested as a potential route for viral entry. Subsequently the same group has demonstrated that F protein not only binds αvβ1 but will interact with a range of integrins known to bind RGD sequences [59]. Although not essential for virus attachment the interaction between the fusion protein and integrins is critical for efficient infection [58,59].

It is still unclear as to whether the binding of hMPV to GAGs is specific, however it is more likely due to non-specific electrostatic interactions and may represent the first step in a multivalent receptor process. In summary, we have shown that diversity exists in GAG binding amongst hMPV G protein lineages and that there is a high affinity interaction between GAGs and the hMPV fusion protein which could explain the dependency of hMPV on glycosaminoglycans.
